# Role of Placental Growth Factor in Predicting Birth Growth in Intrauterine Growth–Restricted Pregnancies and Its Correlation With Placental Histopathology: An Exploratory Study

**DOI:** 10.7759/cureus.98100

**Published:** 2025-11-29

**Authors:** R C Ashwini, Kanya Mukhopadhyay, Nalini Gupta, Naresh Sachdeva, Seema Chopra

**Affiliations:** 1 Pediatrics and Neonatology, Jagadguru Jayadeva Murugarajendra Medical College, Davangere, IND; 2 Pediatrics and Neonatology, Postgraduate Institute of Medical Education and Research, Chandigarh, IND; 3 Cytology and Gynecological Pathology, Postgraduate Institute of Medical Education and Research, Chandigarh, IND; 4 Pediatric Endocrinology, Postgraduate Institute of Medical Education and Research, Chandigarh, IND; 5 Obstetrics and Gynecology, Postgraduate Institute of Medical Education and Research, Chandigarh, IND

**Keywords:** growth at birth, intrauterine growth restriction, neonate, placental growth factor, placental histopathology

## Abstract

Purpose: This study aimed to evaluate the relationship among maternal serum placental growth factor (PlGF), placental histopathology, and intrauterine growth restriction (IUGR).

Methods: This prospective exploratory study was conducted in a Level III neonatal unit between January 2018 and June 2019. Mothers with singleton pregnancies between 30 and 40 weeks of gestation with ultrasound-detected IUGR (n = 41), as well as gestation-matched mothers with normal pregnancies (n = 42), and their neonates were enrolled. Maternal PlGF was measured at the time of IUGR detection in the IUGR group and at comparable gestational ages in the control group admitted with risk for preterm birth. Placental histopathological examination was performed after delivery. Neonatal weight, length, and occipitofrontal circumference were recorded at birth. The outcome measures were maternal PlGF levels, placental histopathology findings in IUGR versus normal pregnancies, and neonatal growth parameters at birth.

Results: At >37 weeks of gestation, the median (IQR) PlGF level was significantly lower in the IUGR group (35 (13, 51) pg/mL) than in the normal pregnancy group (108 (65, 220) pg/mL; p < 0.001), but the difference was not significant at <37 weeks. Maternal PlGF showed a significant positive correlation with birth weight (p = 0.001), length (p = 0.03), occipitofrontal circumference (p = 0.016), and placental histopathology (p = 0.001). Gross placental examination showed lower mean placental weight in IUGR pregnancies compared with normal controls. Features of placental underperfusion due to impaired maternal vascular supply, such as intervillous and perivillous fibrin deposition and foci of calcification, were more common in IUGR placentas than in normal pregnancies.

Conclusions: PlGF levels and placental histopathology were positively correlated with anthropometric measures at birth. Placental weight, placental histopathological changes, and gestational age are significant independent predictors of birth weight and length in IUGR cases.

## Introduction

The burden of intrauterine growth restriction (IUGR) is high in India, where more than 50% of low birth weight (LBW) infants are born with growth retardation [1]. Studies investigating the pathological processes underlying IUGR have shown that abnormal placental function is a common mechanism. However, placental dysfunction often begins gradually and may occur much earlier than clinically detectable IUGR [2]. Differentiating between placentally mediated growth restriction and constitutionally small fetuses remains challenging in routine obstetric practice [3]. Growth restriction may not always be detectable through umbilical artery Doppler, but certain biomarkers may help predict placental insufficiency that leads to fetal growth retardation. There is increasing evidence that an imbalance between proangiogenic factors (e.g., vascular endothelial growth factor (VEGF) and placental growth factor (PlGF)) and antiangiogenic factors (e.g., soluble VEGF receptor-1 (sVEGFR-1) and soluble endoglin (s-Eng)) contributes to the pathophysiology of preeclampsia (PE) and IUGR. Among the maternal serum biomarkers studied, PlGF appears to be the most promising. Therefore, assessing PlGF may help predict intrauterine growth status in mothers with PE.

PlGF is a member of the VEGF family and plays a key role in angiogenesis and trophoblastic invasion of the maternal spiral arteries [4-6]. Several studies, mostly case-control designs, have reported that maternal serum PlGF levels are decreased in pregnancies that deliver small-for-gestational-age (SGA) neonates.

Abnormal histopathological findings in the placentas of IUGR pregnancies may correlate with different clinical outcomes. Villous infarction, villous morphological changes, and maternal vascular abnormalities are frequently observed in placentas from IUGR pregnancies, and these findings may increase the risk of recurrence [7]. This information can influence follow-up and management strategies for affected pregnancies.

We hypothesized that pregnancies complicated by IUGR would have low PlGF levels and abnormal placental histopathology, indicating placental dysfunction, and that these findings may help predict growth outcomes at birth.

## Materials and methods

Study design and setting 

We conducted a prospective exploratory study between January 2018 and June 2019 at a tertiary-level hospital in North India.

Patient Recruitment

All consecutive mothers with singleton pregnancies between 30 and 40 weeks of gestation who were diagnosed with IUGR by ultrasound were prospectively enrolled between January and June 2018 (recruitment phase). Gestational age was determined by first-trimester ultrasonography and/or the date of the last menstrual period. IUGR was defined as a fetal abdominal circumference (AC) below the third percentile for gestational age on antenatal ultrasound [8]. A maternal blood sample (3 mL venous) was collected at enrollment to measure PlGF levels. All enrolled mothers were followed until delivery with abdominal examinations and serial ultrasound biometry. Gestation-matched mothers without IUGR were enrolled simultaneously as controls when admitted with risk for preterm birth, and their blood samples for PlGF were collected at that time. Blood samples were centrifuged at 3000 rpm for 10 minutes and stored at -20°C until analysis. Mothers with congenital fetal malformations, proven intrauterine infection, or multiple pregnancies were excluded. After delivery, placentas were weighed and sent for histopathological examination (HPE). Neonates born to these mothers were enrolled at birth, and nude birth weight was recorded.

Ethical Approval and Consent

This study was approved by the Institute Research Ethics Committee (INT/IEC/2018/000614; dated 02/05/2018). Written informed consent was obtained from all mothers for themselves and their neonates.

Sample Size Estimation

As this was an exploratory clinical study, a non-probability sampling method was used, and a convenience sample of 40 subjects per group was chosen.

Data Collection

Nude birth weight was measured using an electronic scale accurate to ±5 g. Length and occipitofrontal circumference (OFC) were measured at 12-24 hours of age using standard techniques. Z-scores and percentiles before 40 weeks postmenstrual age (PMA) were calculated using Fenton’s method [9] and for term gestation and beyond using the WHO growth standard Anthro Analyzer [10]. Infants below the 10th percentile were classified as having IUGR. All anthropometric measures and baseline characteristics were recorded using a prestructured proforma. Socioeconomic status was assessed using the latest Kuppuswamy scale [11].

PlGF Assay

Frozen maternal samples were thawed and batch-assayed for PlGF using a commercially available kit (DRG International Inc., Springfield, IL, USA). This solid-phase enzyme-linked immunosorbent assay is based on competitive binding as per the manufacturer’s instructions. The detection range was 1.06-1000 pg/mL. The intra-assay coefficient of variation was 2.88%-5.68%, and the inter-assay coefficient of variation was 4.10%-7.00%. Low PlGF was defined as a concentration below the 25th percentile for gestational age, based on PlGF values from normal pregnancies. The technician performing the PlGF analysis was blinded to clinical group allocation.

Placental HPE

Placentas were preserved in 10% formalin and examined by a consultant histopathologist who was blinded to clinical details and neonatal birth weight. Gross examination was performed on the day of delivery and included assessment of (1) cord insertion (central or eccentric), (2) number of cord vessels, (3) membrane insertion (marginal or circumvallate), and (4) microscopic findings. Sections were stained with hematoxylin and eosin. Microscopy assessed villous immaturity, villitis, villous fibrosis, placental hypoplasia, thrombotic vasculopathy, perivillous fibrin deposition, retroplacental clot, infarctions (location, single/multiple), intervillous hemorrhage and thrombosis, hemorrhagic endovasculitis, calcification, fibrinoid necrosis, leucocytic infiltration, villous edema, erythroblastosis, increased syncytial knots, chorioamnionitis, and chorangiosis. The presence of any one abnormality was considered abnormal.

Statistical Analysis

Statistical analysis was performed using IBM SPSS Statistics for Windows, Version 24.0 (IBM Corp., Armonk, NY, USA). Comparisons were made using independent t-tests or chi-square tests, as appropriate. Spearman’s correlation coefficient was used for skewed data to assess positive or negative correlations. A p-value < 0.05 was considered statistically significant. Low PlGF was defined as a concentration below the 25th percentile for gestational age. Sensitivity, specificity, positive predictive value (PPV), and negative predictive value (NPV) were calculated for cutoff values. Multivariate analysis and regression were performed to assess predictors (gestational age, sex, placental weight, and PlGF) for birth weight <10th percentile and birth length.

## Results

Study flow and baseline characteristics

**Figure 1:**
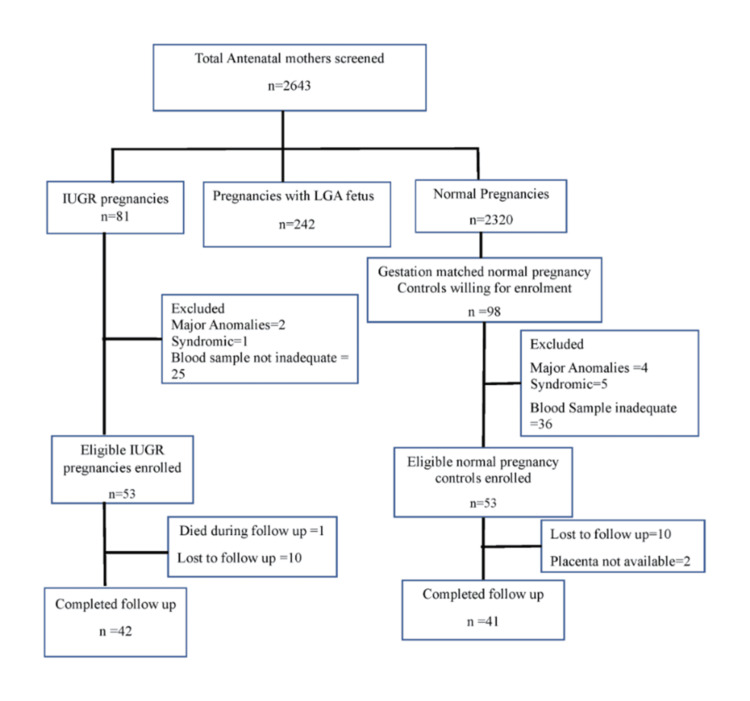
Flowchart summarizing the enrollment of study subjects Out of 2643 antenatal mothers screened, 81 had IUGR, 242 had LGA pregnancies, and 2320 had normal pregnancies. Of these, 53 mothers with IUGR were eligible for enrollment. Ninety-eight gestation-matched controls consented to participate, of whom 45 were excluded, leaving 53 eligible controls. One mother in the IUGR group died. Among the remaining participants (IUGR n = 52; AGA n = 53), 10 mothers in each group were lost to follow-up, and two placentas from the normal pregnancy group were discarded and unavailable for final analysis. Therefore, 42 mothers in the IUGR group and 41 in the normal pregnancy group were included in the final analysis. IUGR: intrauterine growth restriction, LGA: large for gestational age, AGA: appropriate for gestational age.

A total of 42 mothers with antenatal diagnoses of IUGR and their neonates, and 41 gestation-matched mothers with normal pregnancies and their neonates, were included in the final analysis. A flowchart of the study participants is shown in Figure 1.

The baseline characteristics of the IUGR and appropriate-for-gestational-age (AGA) infants are presented in Table 1. The mean gestational age at enrollment for mothers with IUGR pregnancies was 35.9 (2.4) weeks. The mean (SD) birth weight and gestational age of the enrolled IUGR infants were 1879 (528) g and 36.5 (2.4) weeks, respectively. In comparison, the mean (SD) birth weight and gestational age of the AGA infants were 2870 (590) g and 36.2 (2.2) weeks, respectively.

**Table 1:** Maternal and neonatal baseline demographic variables IUGR: intrauterine growth restriction, AGA: appropriate for gestational age, SES: socioeconomic score, GA: gestational age, PIH: pregnancy-induced hypertension, GDM: gestational diabetes mellitus. ^a^n (%). ^b^Mean (SD).

Variable	IUGR pregnancy (n = 42)	Normal pregnancy (n = 41)
Maternal variables		
Maternal age in years^a^	26.4 (6)	26.7 (10)
Primigarvida^a^	20 (47.6)	22 (53.7)
SES total score^a^	3.0 (0.9)	2.6 (0.7)
Maternal illness		
GDM^b^	1 (2.4)	7 (17.1)
Hypothyroidism^b^	8 (19)	10 (24.4)
Anemia^b^	7 (16.7)	2 (4.9)
PIH^b^	15 (35.7)	4 (9.8)
GA at enrolment in weeks^a^	35.9 (2.4)	36.5 (2.2)
Mode of delivery: vaginal route	24 (57.1)	21 (51.2)
GA at delivery in weeks^b^	36.5 (2.4)	36.6 (2.2)
Placental variables		
Placental weight (g)^ b^	380(80.8)	502.7(131.5)
Placental weight percentile: <10th centile^a^	31 (73.8)	16 (39)

Maternal PlGF Levels in Term and Preterm Normal and IUGR Pregnancies

The median (IQR) PlGF level in the IUGR group at <36+6 weeks’ gestation was 33 (12.3, 88.6) pg/mL, which was lower but not significantly different from the AGA group (42.1 (27, 50.8) pg/mL; p = 0.69). At >37 weeks’ gestation, the median (IQR) PlGF level in the IUGR group was 35 (13, 51) pg/mL, which was significantly lower than that in the AGA group (108 (65, 220) pg/mL; p < 0.001).

Percentile values for PlGF in preterm and term normal pregnancies were calculated (Table 2). Values below the 25th percentile (27.3 pg/mL in preterm pregnancies and 65.8 pg/mL in term pregnancies) were used as cutoff levels for low PlGF to identify IUGR pregnancies.

**Table 2:** Percentiles of maternal PlGF levels in normal term and preterm pregnancies PlGF: placental growth factor.

Normal pregnancy			
Percentile of maternal PlGF	Preterm (n = 15) (pg/mL)	Term (n = 26) (pg/mL)	Combined (n = 41) (pg/mL)
5th	16	26.7	16.9
10th	16.1	34.2	24.3
25th	27.3	65.8	37.2
50th	42.1	108.8	82.7
75th	50.8	220.3	118.3
90th	111.1	236.3	229.3
95th	-	246.4	241

The diagnostic ability of PlGF to detect IUGR pregnancy at < 25th percentile (cutoff of 27.3 pg/mL in preterm pregnancy and 65.8 pg/mL in term pregnancy) was assessed, as depicted in Table 3. In the preterm group, the <25th percentile cutoff showed low sensitivity of 43% and 80% specificity. In contrast, toward term gestation, sensitivity and specificity were high (84% and 77%, respectively). Cutoffs 27.3 pg/mL in preterm pregnancy and 65.8 pg/mL in term pregnancy had a PPV of 70% and 77%, respectively.

**Table 3:** Diagnostic ability of maternal serum PlGF for detecting IUGR status at birth LR^+^: positive likelihood ratio, LR^-^: negative likelihood ratio, PPV: positive predictive value, NPV: negative predictive value, PlGF: placental growth factor, IUGR: intrauterine growth restriction.

Cutoff by percentile method (for detecting IUGR at birth <25th percentile cutoff)		
	27.3 pg/mL (preterm)	65.8 pg/mL (term)
Sensitivity	0.43	0.84
Specificity	0.80	0.77
LR^+^	2.1	3.6
LR^-^	0.7	0.21
PPV, %	70	77
NPV, %	57	83

**Figure 2:**
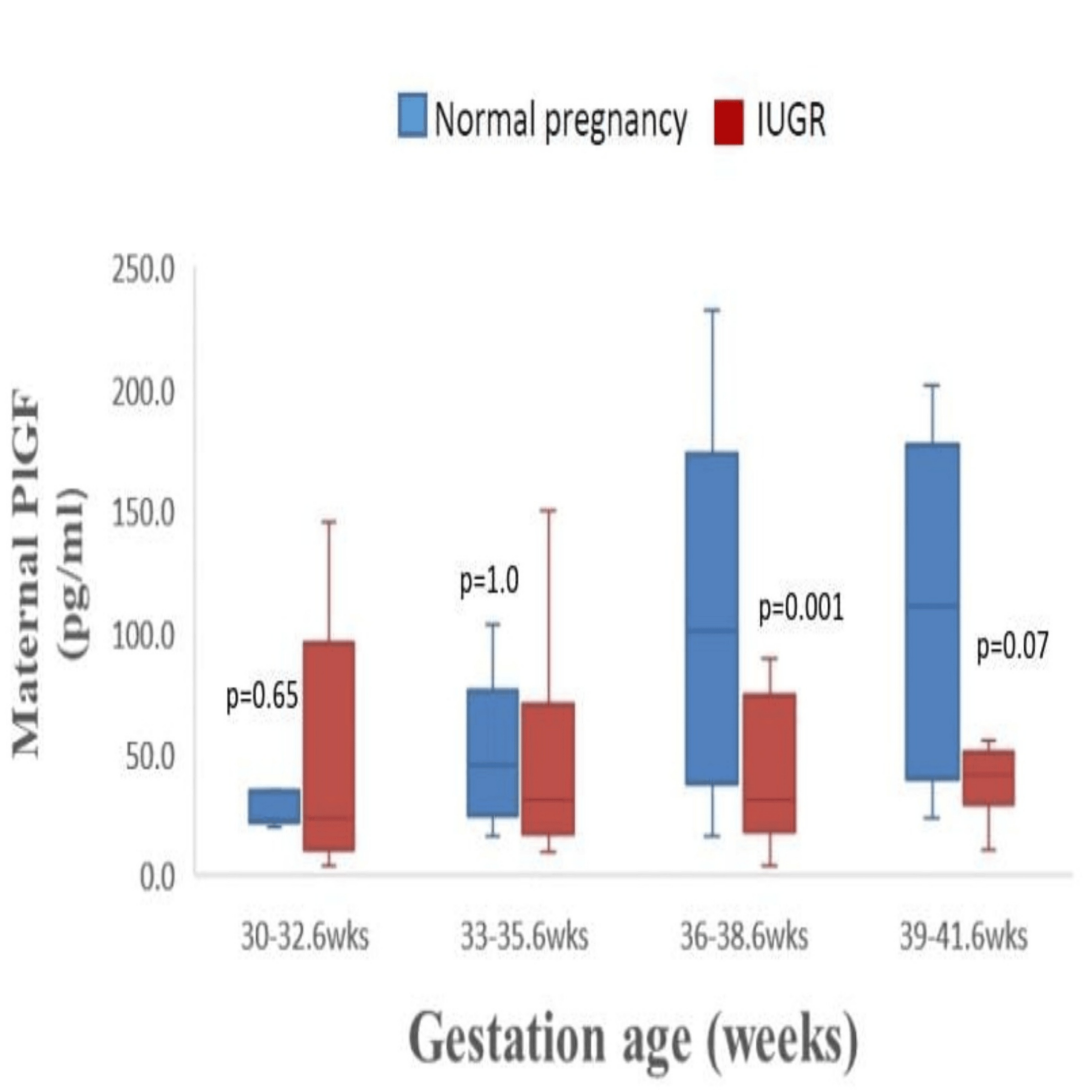
Box and whisker plots depicting comparisons of maternal serum PlGF levels at different gestational age groups in normal and IUGR pregnancies PlGF: placental growth factor, IUGR: intrauterine growth restriction.

Maternal PlGF levels were higher in normal pregnancies than in the IUGR group (Figure 2) for gestational ages > 33 weeks, with statistically significant differences (p < 0.001) observed in the 36 to 38+6-week subgroup. Due to the small number of mothers enrolled at <32 weeks, this difference could not be reliably assessed in earlier gestations.

PlGF and Abnormal Placental Histopathology in IUGR Pregnancy

Median (IQR) maternal PlGF levels were lower in the IUGR group (33.2 pg/mL (12.5, 82.8)) compared with normal pregnancies (82.7 pg/mL (7.2, 118.3)). The mean (SD) placental weight was also lower in the IUGR group than in normal pregnancies (380 g (80.8) vs. 502 g (131.5); p < 0.001). Placental histopathological findings in the IUGR and normal pregnancy groups are presented in Table 4.

**Table 4:** Comparison of maternal PlGF, gross examination, and placental histopathological examination findings in IUGR and normal pregnancy groups IUGR: intrauterine growth restriction, PlGF: placental growth factor. ^$^Median (IQR). ^^^Mean (SD). ^#^n (%). *p < 0.05 is statistically significant.

	IUGR group (n = 42)	Normal pregnancy group (n = 41)	p-value
Maternal PlGF (pg/mL)^$^	33.2 (12.5, 82.8)	82.7 (7.2, 118.3)	<0.001*
Gross examination findings			
Placenta weight (g)^	380 (±80.8)	502 (±131.5)	<0.001*
Eccentric cord insertion	6 (14)	0	0.05
Microscopic findings^#^			
Maternal vascular supply obstruction			
Intervilllous fibrin deposition	12 (28)	7 (17)	<0.001*
Perivillous fibrin deposition	17 (40)	7 (17)	<0.001*
Foci of calcification	22 (52)	5 (12)	<0.001*
Foci of infarction	5 (12)	0	<0.001*
Syncytial knots	3 (7)	0	<0.001*
Intervillous hemorrhage	1 (2.5)	0	<0.001*
Fetal vascular supply obstruction			
Thrombotic vasculopathy	3 (7)	0	<0.001*

Maternal vascular supply obstruction, including intervillous and perivillous fibrin deposition, foci of calcification, foci of infarction, syncytial knots, and intervillous hemorrhage, was the most prominent placental histopathological finding in IUGR pregnancies. Thrombotic vasculopathy suggestive of fetal vascular supply obstruction was observed in 7% of IUGR cases but was not seen in normal pregnancies.

**Figure 3:**
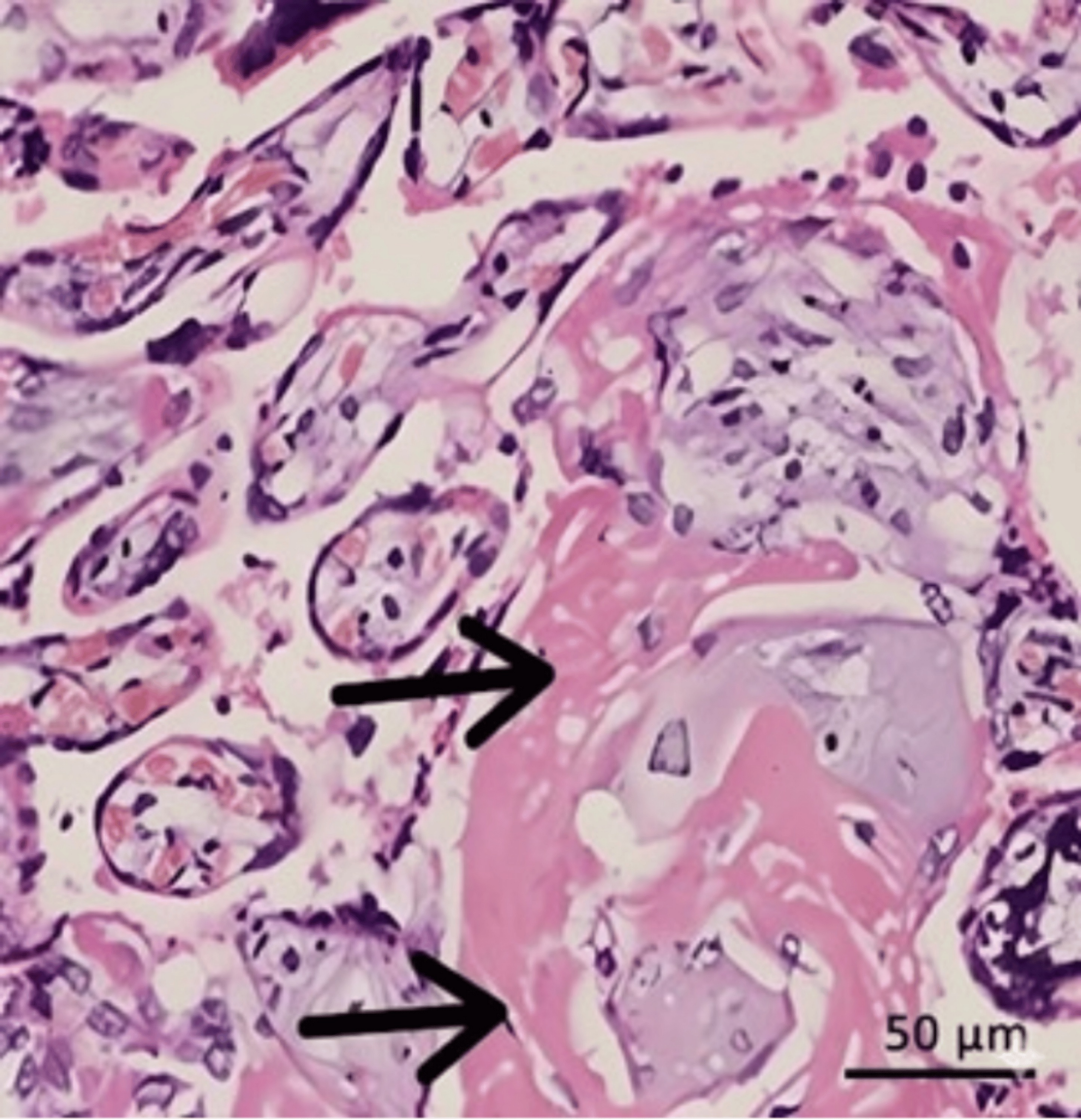
Section of placental tissue from an IUGR pregnancy stained with H&E (400×), showing increased perivillous fibrin deposition (black arrows) IUGR: intrauterine growth restriction, H&E: hematoxylin and eosin staining.

**Figure 4:**
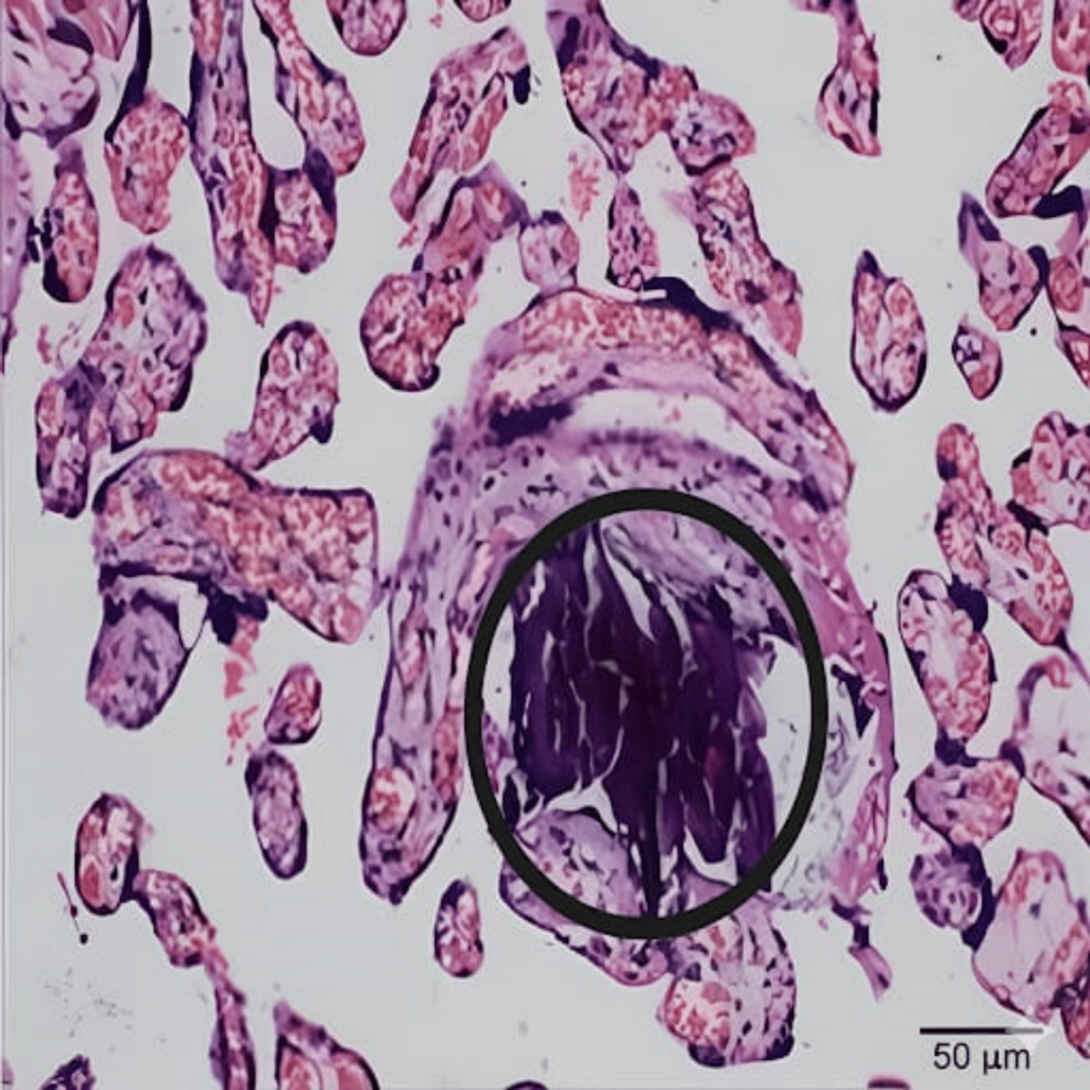
Section of placental tissue from an IUGR pregnancy stained with H&E (400×), showing extensive calcification (circled) IUGR: intrauterine growth restriction, H&E: hematoxylin and eosin staining.

In the IUGR group, features of placental underperfusion due to maternal vascular obstruction, such as intervillous and perivillous fibrin deposition (Figure 3), were the most common findings. The next most frequent finding was foci of calcification (Figure 4) in the placental histopathology.

Prediction of Birth Anthropometry Based on PlGF and Placental HPE

A multiple regression model was performed to assess whether maternal pregnancy-induced hypertension (PIH), serum PlGF levels, gestational age at birth, sex, placental weight, and placental histopathological changes could predict birth weight. The model showed that placental weight (B = 1.30, t = 3.16, p < 0.001; 95% CI: 0.28-4.97) and gestational age at birth (B = 170.5, t = 1.51, p < 0.001) were significant predictors of birth weight. The overall model was significant (F(6, 76) = 21.31, p < 0.001) and explained 62.7% of the variance in birth weight (R² = 0.627). Thus, placental weight and gestational age at birth were identified as independent predictors of neonatal birth weight.

Correlation Between PLGF and Birth Anthropometry and Abnormal Placental HPE 

We found a significant positive correlation between maternal serum PlGF levels and birth weight (R = 0.37, p = 0.001), birth length (R = 0.24, p = 0.03), and occipitofrontal circumference (OFC) at birth (R = 0.26, p = 0.016).

There was also a significant association between abnormal placental histopathological findings and reduced anthropometric measurements at birth (p < 0.001). Low PlGF levels were positively correlated with abnormal placental histopathology (R = 0.35, p = 0.001).

## Discussion

Our study confirmed that maternal serum PlGF levels are significantly lower in pregnancies complicated by fetal growth restriction (FGR) compared to normal pregnancies. Using the percentile method, we identified a cutoff level (<25th percentile: 27.3 pg/mL at <37 weeks and 65.8 pg/mL at ≥37 weeks) to detect IUGR at birth. Based on this cutoff, the sensitivity, specificity, PPV, and NPV of PlGF for identifying IUGR were higher in neonates ≥37 weeks than in those <37 weeks. We also observed a significant positive correlation between maternal serum PlGF and birth weight, birth length, and OFC. Reduced PlGF in IUGR pregnancies was associated with poorer fetal outcomes, including LBW, smaller head circumference, and shorter length. Low PlGF in IUGR pregnancies also correlated with the most common placental histopathology pattern, maternal vascular supply obstruction, which reflects impaired placental angiogenesis and perfusion.

Parchem et al. [12] similarly considered <12 pg/mL as very low and <100 pg/mL as low PlGF and found that women with low PlGF delivered SGA neonates.

Ashwal et al. defined low PlGF as <10th percentile for gestational age, compared to our <25th percentile cutoff [13]. Their findings aligned with ours: low PlGF was associated with higher rates of IUGR (8.3 vs 42.9 pg/mL, p = 0.001). They also reported that low PlGF was strongly associated with maternal vascular malperfusion on placental histopathology (72.7%, p < 0.001).

Raghavendra et al. [14] and Meena et al. [15] reported that pregnant women with PlGF <100 pg/mL had an increased risk of SGA neonates compared to those with normal PlGF levels.

McLaughlin et al. showed that PlGF < 100 pg/mL was linked with a higher risk of delivering neonates with birth weight < 10th centile (OR 6.4 (4.6-8.8)) [16]. Similar to our findings, they observed that low PlGF was associated with maternal vascular malperfusion in 77% of cases, fetal thrombotic vasculopathy in 25%, followed by chronic intervillositis, villitis of unknown origin, and perivillous fibrin deposition [16]. Fillion et al. [17] also demonstrated a strong correlation between low PlGF and maternal vascular malperfusion, even in the absence of preeclampsia.

Audette et al. reported that low PlGF was associated with lower placental weight (447 vs 471 g, p = 0.01), abnormal cord insertion (25% vs 12%, p = 0.001), and histopathological evidence of maternal vascular malperfusion (18% vs 11%, p = 0.04) [18].

Often, no clear cause is identified in late-onset IUGR among mothers without PE. In our study, low PlGF in IUGR pregnancies correlated with the most common placental abnormality, maternal vascular supply obstruction, highlighting impaired placental angiogenesis and perfusion and supporting the concept of “placental IUGR.” Consistent with our findings, Shinar et al. [19] reported that pregnancies with low PlGF were more likely to have an estimated fetal weight < 5th centile (73.8% vs 53%, p = 0.01). Low PlGF identified maternal vascular malperfusion with a sensitivity of 70.1% (58.6-80.0) and specificity of 79.6% (64.7-90.2), with an AUC of 0.73 (0.63-0.84). PlGF outperformed other sonographic markers of placental FGR, both alone and in combination.

The strengths of our study include being the first in India to examine maternal serum PlGF in IUGR pregnancies and its association with birth outcomes and placental pathology. Western literature has not reported long-term outcomes in cases where PlGF was assessed. Since this is an exploratory study, multiple comparisons were made to identify possible associations.

The limitations of our study include the small sample size and the absence of standardized cutoff values for PlGF. Kit sensitivity varies, and each laboratory must establish its own cutoff using standard curves provided by the manufacturer. Additionally, PlGF levels change throughout gestation, which can complicate the interpretation of what constitutes a true positive or negative value when diagnosing IUGR at a single time point.

## Conclusions

Maternal serum PlGF showed a significant positive correlation with birth anthropometric measures and with abnormal placental histopathology in pregnancies affected by IUGR. Because PlGF demonstrated higher sensitivity at term, a cutoff value of 65.8 pg/mL at 37 weeks’ gestation appears to be a useful non-invasive biomarker for confirming placentally mediated IUGR. Incorporating maternal serum PlGF into management algorithms for late-onset growth restriction may help clinicians more accurately identify high-risk pregnancies. In women with low PlGF, closer antenatal monitoring and improved care may reduce the risk of further growth restriction. Mean placental weight was lower in pregnancies with IUGR, and abnormal placental histopathological findings were positively correlated with poorer anthropometric outcomes at birth. Although IUGR neonates are often evaluated after delivery to determine the cause, many cases remain idiopathic. Placental underperfusion as an etiology is likely underrecognized; therefore, routine placental histopathological examination is clinically relevant and valuable for confirming placental IUGR.

Future research should focus on evaluating PlGF together with biophysical parameters and maternal characteristics to improve the prediction of IUGR. Establishing gestational age-specific reference nomograms for PlGF levels in the Indian population would also support more accurate and standardized clinical use.
